# Virtual Resection Specimen Interaction Using Augmented Reality Holograms to Guide Margin Communication and Flap Sizing

**DOI:** 10.1002/ohn.325

**Published:** 2023-03-19

**Authors:** Fabian N. Necker, Marcello Chang, Christoph Leuze, Michael C. Topf, Bruce L. Daniel, Fred M. Baik

**Affiliations:** 1 Department of Radiology, Incubator for Medical Mixed Reality at Stanford (IMMERS), Stanford University, Palo Alto, California, USA; 2 Institute of Functional and Clinical Anatomy, Faculty of Medicine, Friedrich-Alexander-Universität Erlangen-Nürnberg (FAU), Erlangen, Germany; 3 Department of Otolaryngology–Head and Neck Surgery, Vanderbilt University Medical Center, Nashville, Tennessee, USA; 4 Department of Otolaryngology–Head and Neck Surgery, Stanford University School of Medicine, Stanford, California, USA

**Keywords:** 3D scanning, augmented reality, computer-aided surgery, computer-guided surgical planning, digital anatomy, digital pathology, head-neck cancer, head-neck surgery, HoloLens, microvascular reconstruction

## Abstract

Head and neck surgeons often have difficulty in relocating sites of positive margins due to the complex 3-dimensional (3D) anatomy of the head and neck. We introduce a new technique where resection specimens are 3D scanned with a smartphone, annotated in computer-assisted design software, and immediately visualized on augmented reality (AR) glasses. The 3D virtual specimen can be accurately superimposed onto surgical sites for orientation and sizing applications. During an operative workshop, a surgeon using AR glasses projected virtual, annotated specimen models back into the resection bed onto a cadaver within approximately 10 minutes. Colored annotations can correspond with pathologic annotations and guide the orientation of the virtual 3D specimen. The model was also overlayed onto a flap harvest site to aid in reconstructive planning. We present a new technique allowing interactive, sterile inspection of tissue specimens in AR that could facilitate communication among surgeons and pathologists and assist with reconstructive surgery.

Intraoperative consultation of pathologists via frozen section analysis is a critical part of head-neck oncologic surgery.^1^ Communication of margin results can be challenging and continues to be dependent upon verbal descriptions via telephone, which can lead to miscommunication.^2^

Here, we describe our technique for virtual resection specimen interaction using augmented reality (AR) holograms. Three-dimensional (3D) holograms can be created using modern smartphones, and in combination with AR glasses, may allow more intuitive visualization and inspection of pathologists’ findings using annotations on this 3D model. The surgeon can easily manipulate these 3D specimen holograms in a sterile environment with only their hands and can be used to guide margin communication between surgeon and pathologist as well as for reconstructive planning.

## Subjects and Methods

**Figure 1.**
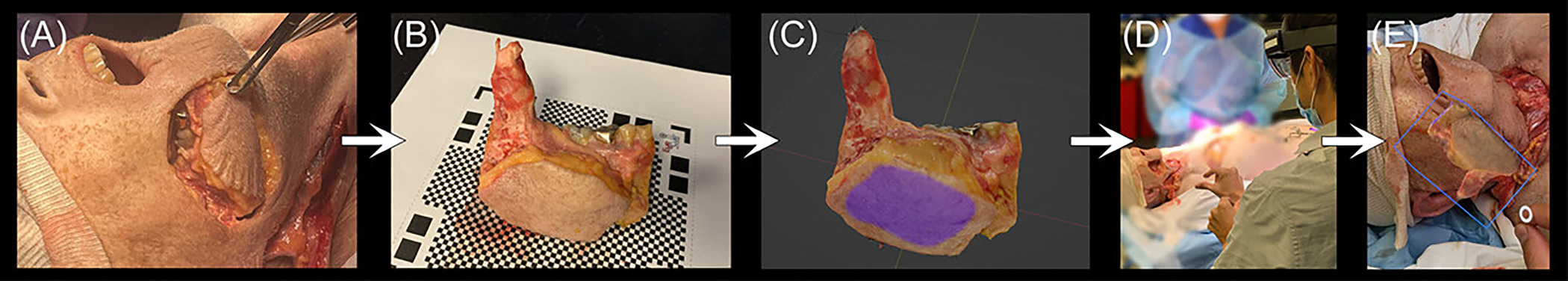
Overview of the entire workflow for the mandible model. (A) Resection still be in its in situ position. (B) Removed tissue resection on the scanning mat. (C) A 3D-scanned model with color-coded surface annotations. (D) The surgeon positioned the virtual model back onto the cadaver. (E) In situ superimposed annotated model back in the body, as seen through the AR glasses. 3D, 3-dimensional; AR, augmented reality.

**Figure 2.**
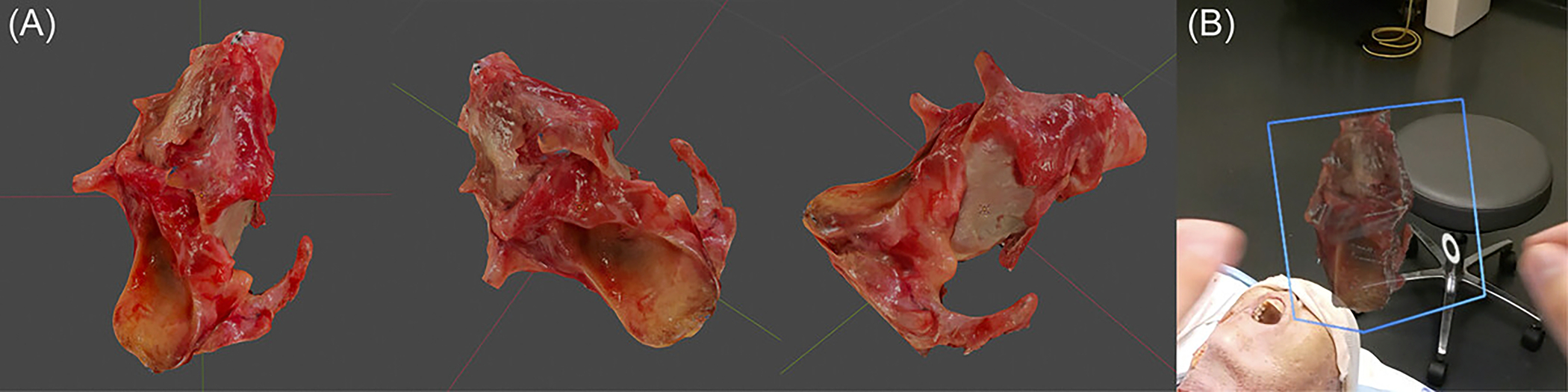
Virtual, 3-dimensional-scanned larynx resection model. (A) Model inspected from different perspectives. (B) Model as viewed through the HoloLens.

We performed 2 common oncologic resections (Figures 1 and 2) on cadaver specimens, provided through the Stanford Clinical Anatomy department. Permission to use the following media was obtained. An ethics review from the Institutional Review Board was not necessary for cadaver studies as per Stanford Research Compliance guidelines.

3D scans of specimens were acquired using an Apple iPhone XS and the 3D scanning app Qlone (Qlone; EyeCue Vision Technologies). Qlone has been used to capture cadaveric specimens in neurosurgery^3^ and produces consistent results for reflective objects such as our resected specimens.

For 3D scanning, we placed the tissue sample on a checkerboard-like mat and acquired images of the tissue sample covering a half-dome shape around the sample (Figure 1B). We used semitransparent ink in Blender (Blender Foundation) to annotate and color-code anatomical orientations on the model surfaces to imitate what could become a specialized pathology staining protocol (Figure 1C) (Supplemental Video 1, available online). Such annotations are similar to previous 3D scan studies in head-neck surgery.^4^

**Figure 3.**
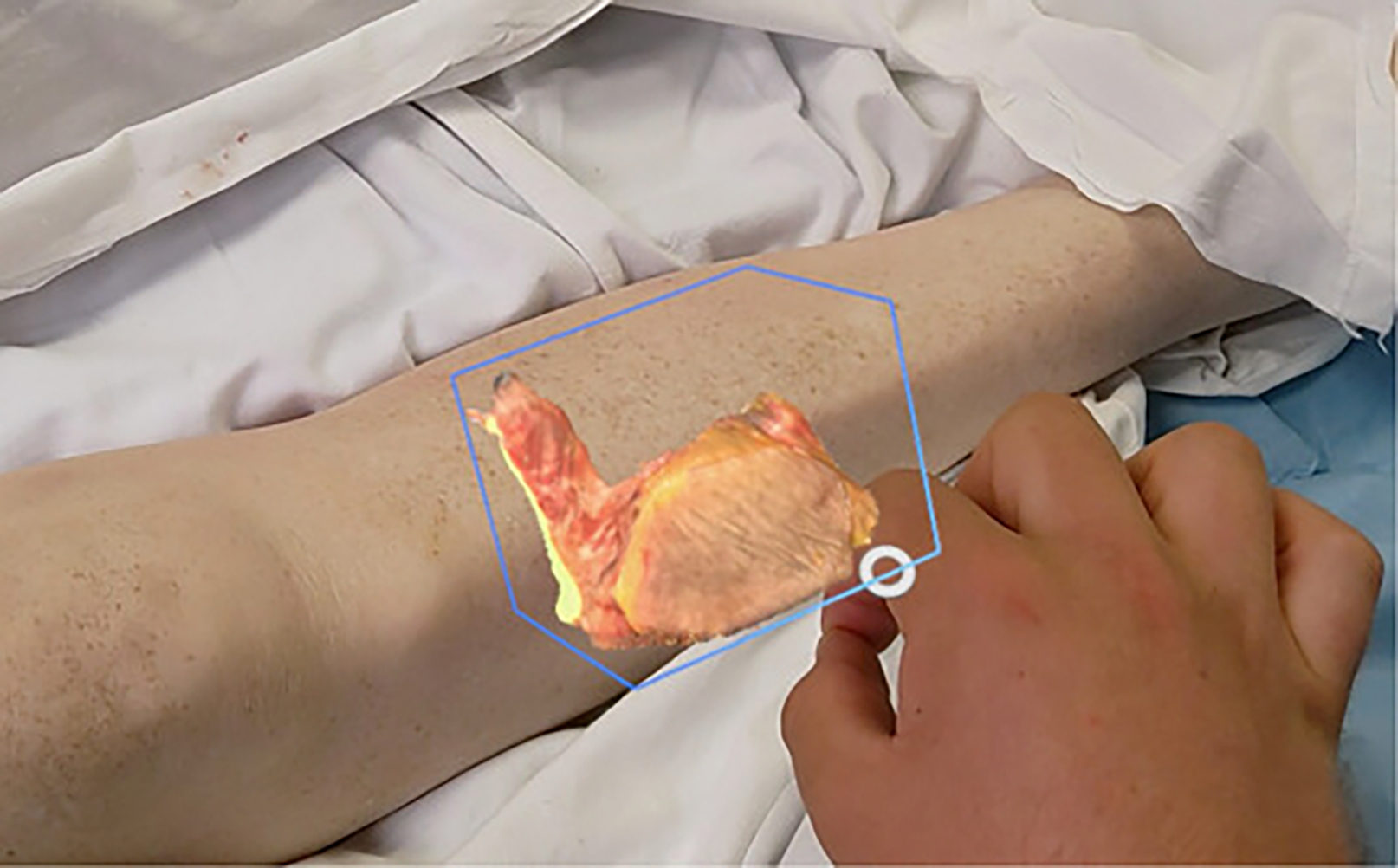
Virtual, 3-dimensional-scanned mandible resection model moved to lower leg size persistently. Flap size estimation could be performed by having the original resection as a reference.

The surgeon then donned the see-through AR headset HoloLens 2 and started the Microsoft Mesh application (Microsoft), a collaborative 3D workspace. Here, users can view and manipulate these 3D models while still being able to see their real-world surroundings. Within Mesh, the surgeon then resized the annotated specimen model using intuitive, sterility-preserving hand gestures (Figure 1D). Finally, the surgeon positioned the virtual 3D model back at its original in situ location (Figure 1E) and a potential flap-harvest site (Figure 3).

## Results

During an operative training workshop, a mandibulectomy (Figure 1) and laryngectomy (Figure 2) were performed and the specimens were scanned. The entire processing time from the 3D scan to the final 3D visualization on the HoloLens was under 10 minutes. The surgeon scaled up the virtual 3D model for close-up inspection and scaled it back down to be accurately placed in its original in situ position on the cadaver’s defect sites (Figure 1E). Subsequently, the surgeon moved the model to a fibula harvest site (Figure 3) maintaining the same size as when fitted in the defect. Overlay of the resection specimen allowed for accurate guidance of flap dimensions to be harvested. The entire process is demonstrated in Supplemental Video 2, available online.

## Conclusion

To our knowledge, this report is the first to demonstrate the 3D in-space visualization and manipulation of a virtual resection specimen in mixed reality. Superimposing the virtual resection model back in the original defect site allowed the surgeon an intuitive understanding of the orientation. Referring to anatomical directions with color coding of relevant surfaces made communication easier, which may allow surgeons to identify the precise location of positive margins more accurately.

Transporting a size-persistent 3D model to flap harvest sites could also be beneficial in soft-tissue reconstruction. In this scenario, the virtual model scaled to the original size could be used as an outline to draw the exterior border of a tissue flap more accurately with a marker on the harvest site. Additionally, for bony reconstruction, this mixed-reality platform may be utilized to overlay a preplanned model over the surgical bed to guide osteotomies and confirm the proper alignment of bone segments.

A complex topic warranting further investigation is mucosal shrinkage. As perfusion stops, mucosa specimens can shrink significantly (10%−47%) in oral cavity resections.^5,6^ Using computer-assisted design software, the scan could be artificially enlarged by tissue-defined percentages. However, this is highly variable depending on tissue type and would need further investigation, especially as advanced tumors (T3/T4) have been reported to shrink less and more irregularly.^7^

Our technique would also be beneficial for pathologists coming to the operating room (OR) in person to inspect the specimen in vivo prior to resection. A real-time collaborative 3D meeting afterward where the surgeon and pathologist share the same hologram in space would enable the pathologist to see the surgeon’s realignment of the virtual annotated specimen facilitating easier discussion of findings. Furthermore, this technique creates an accurate, nondestructive 3D log of the specimen, which may more effectively communicate specimen margins to radiation oncologists, medical oncologists, and other pathologists.

The entire technique can be performed quickly (under 10 minutes) and cost-effectively. Only AR glasses are additionally needed (HoloLens ca. $3500, with other models currently on the market being more affordable). Current limitations include the accuracy of the scan, which could be addressed by using a commercial desktop 3D scanner that has been validated for specimens in head-neck surgery.^8^ Additionally, the accuracy and stability of the 3D specimen hologram warrant further investigation when translating this technique into the OR.

We believe that these case examples lay the foundation for the future use of AR specimen models. We envision that these models can significantly improve intraoperative workflow in head and neck surgery for pathologists and surgeons alike.

## Supplementary Material

Supplementary Video 1

Supplementary Video 2

Additional supporting information is available in the online version of the article.
